# Similar Source of Differential Blood mRNAs in Lung Cancer and Pulmonary Inflammatory Diseases: Calls for Improved Strategy for Identifying Cancer-Specific Biomarkers

**DOI:** 10.1371/journal.pone.0108104

**Published:** 2014-09-22

**Authors:** Guini Hong, Beibei Chen, Hongdong Li, Wenjing Zhang, Tingting Zheng, Shan Li, Tongwei Shi, Lu Ao, Zheng Guo

**Affiliations:** 1 Bioinformatics Centre, School of Life Science, University of Electronic Science and Technology of China, Chengdu, China; 2 Department of Bioinformatics, School of Basic Medical Sciences, Fujian Medical University, Fuzhou, China; 3 College of Bioinformatics Science and Technology, Harbin Medical University, Harbin, China; Harbin Medical University, China

## Abstract

**Background:**

Many studies try to identify cancer diagnostic biomarkers by comparing peripheral whole blood (PWB) of cancer samples and healthy controls, explicitly or implicitly assuming that such biomarkers are potential candidate biomarkers for distinguishing cancer from nonmalignant inflammation-associated diseases.

**Methods:**

Multiple PWB gene expression profiles for lung cancer/inflammation-associated pulmonary diseases were used for differential mRNAs identification and comparison and for proportion estimation of PWB cell subtypes.

**Results:**

The differentially expressed genes (DE genes) between lung cancer/inflammation-associated pulmonary patients and healthy controls were reproducibly identified in different datasets. For these DE genes observed in lung cancer/inflammation-associated pulmonary diseases, more than 90.2% were differentially expressed between myeloid cells and lymphoid cells, with at least 96.8% having consistent directions of regulation (up- or down-regulations) in myeloid cells compared to lymphoid cells, explainable by the shifted populations of PWB cell subtypes under the disease conditions. The comparison of DE genes for lung cancer and inflammation-associated pulmonary diseases showed that the overlapping genes were 100% consistent in the sense of direction of regulation.

**Conclusions:**

The differential blood mRNAs observed in lung cancer and in inflammation-associated pulmonary diseases were similar, both mainly reflecting the difference between myeloid cells and lymphoid cells predominantly determined by PWB cell population shifts. Thus, the strategy of comparing cancer with healthy controls may provide little information of the ability of the identified candidate biomarkers in discriminating cancer from inflammation-associated pulmonary diseases.

## Introduction

Lung cancer is one of the most prevalent cancer types [1]. Early detection of lung cancer is crucial to avoid treatment delay and improve survival. To find diagnostic biomarkers of lung cancer for non-invasive clinical application, researchers have extensively studied gene expression changes in peripheral whole blood (PWB) or peripheral blood mononuclear cells (PBMCs) [2]–[6]. Although some blood-based gene expression signatures have been reported to have good performance in discriminating lung cancer from healthy controls [2]–[4], they usually lack reproducibility between laboratories. More importantly, few studies have evaluated whether the identified blood-based gene expression signatures have the ability to distinguish lung cancer from inflammation-associated diseases of the lung, including but not limited to sarcoidosis, pneumonia and tuberculosis, which have similar clinical and histological features with lung cancer [6].

It is assumed that peripheral leukocytes are the dominating source of the mRNA in PWB samples [7]; however, the differential mRNA signals observed in PWB samples from cancer patients compared to healthy controls may reflect changes in the subsets in peripheral blood cells. Many studies have found that, in PWB of patients with cancer, the proportion of blood cells originated from the myeloid progenitor (referred to as myeloid cells for simplicity) increases, while the proportion of blood cells originated from the lymphocyte progenitor (referred to as lymphoid cells for simplicity) decreases [8]–[13]. The proportional changes (or subpopulation shifts) of the cell types in PWB could affect the gene expression profiles of cancer PWB samples compared to healthy controls [14], [15]. However, to what extent the changes in blood cell populations contribute to the differential gene expression changes observed in lung cancer PWB samples is still unclear. As a compounding factor, similar subpopulation shifts in blood cells have also been observed in many inflammation-associated pulmonary diseases [16]–[18]. Therefore, the elucidation of the source of the differential mRNAs in PWB samples of inflammation-associated pulmonary diseases is needed to assess whether the gene expression signatures determined from lung cancer PWB compared to healthy controls have the ability to distinguish cancer from other inflammation-associated pulmonary diseases.

In this study, using three gene expression profiles of PWB samples from lung cancer patients, we showed that the differentially expressed genes (DE genes) detected from different datasets were significantly reproducible. By applying a deconvolution algorithm, we showed that the proportion of myeloid cells increased and the proportion of lymphoid cells decreased in lung cancer PWB samples. We further showed that the DE genes between PWB samples of lung cancer and healthy controls were highly consistent with the DE genes between myeloid cells and lymphoid cells, supporting the possibility that the differential mRNAs observed in lung cancer PWB samples were defined by differential mRNAs between myeloid cells and lymphoid cells in PWB samples of lung cancer patients. Especially, the most pronounced DE genes between PWB samples of lung cancer and healthy controls tended to be defined by DE genes between myeloid cells and lymphoid cells. The same phenomena were observed for various inflammation-associated pulmonary diseases. Therefore, it could be difficult to use PWB gene expression signatures developed from lung cancer versus healthy controls as potential candidate biomarkers to distinguish cancer from inflammation-associated pulmonary diseases. To develop specific diagnostic biomarkers for cancer, future studies might focus on the direct comparison between blood-based gene expression profiles of PWB cell subtypes between cancer and inflammation-associated disease.

## Materials and Methods

### Analysis of microarray data

We analyzed three microarray datasets for PWB samples from each type of pulmonary diseases (Table 1), including lung cancer, sarcoidosis, pneumonia and tuberculosis. All of the gene expression datasets analyzed in this study were downloaded from the Gene Expression Omnibus (GEO) database [19]. For simplicity, sarcoidosis, pneumonia and tuberculosis are also referred to as “inflammation-associated pulmonary diseases”. Samples in LC60, SCD68, PNU58 and TB63 were extracted from the GEO series GSE42826 while samples in LC46, SCD55, PNU46 and TB54 were extracted from the GEO series GSE42830. Expression data from different studies for lung cancer and each inflammation-associated pulmonary disease, namely the LC153, SCD58, PNU26 and TB83, were also collected for evaluation of reproducibility. The normalized data were downloaded from GEO, and the original platform annotation file obtained from GEO for each dataset was used to annotate the CloneIDs to GeneIDs.

**Table 1:** Datasets analyzed in this study.

Phenotype	Dataset^a^	Reference	Case:Control^b^	GEO acc No^c^	Platform	No. of genes
Lung	LC60	Bloom [6]	8∶52	GSE42826	GPL10558	30500
	LC46	Bloom [6]	8∶38	GSE42830	GPL10558	30500
	LC153	Rotunno [4]	73∶80	GSE20189	GPL571	12790
Sarcoidosis	SCD68	Bloom [6]	16∶52	GSE42826	GPL10558	30500
	SCD55	Bloom [6]	17∶38	GSE42830	GPL10558	30500
	SCD58	Koth [37]	38∶20	GSE19314	GPL570	20283
Pneumonia	PNU58	Bloom [6]	6∶52	GSE42826	GPL10558	30500
	PNU46	Bloom [6]	8∶38	GSE42830	GPL10558	30500
	PNU26	Koth [37]	6∶20	GSE19314	GPL570	20283
Tuberculosis	TB63	Bloom [6]	11∶52	GSE42826	GPL10558	30500
	TB54	Bloom [6]	16∶38	GSE42830	GPL10558	30500
	TB83	Maertzdorf [38]	46∶37	GSE28623	GPL4133	19751
Leukocyte cells	LEU33	Allantaz [20]	13∶20	GSE28491	GPL570	10698
	LEU37	Allantaz [20]	17∶20	GSE28490	GPL570	11241

a Each dataset is denoted by the following nomenclature: phenotype followed by the sample number,

b The number of case and control samples. For leukocyte cells, Case refers to myeloid group; Control refers to lymphoid group,

c GEO accession number. All the microarray data are accessible through NCBI's Gene Expression Omnibus (GEO, http://www.ncbi.nlm.nih.gov/geo) with the corresponding GEO accession number.

The two leukocyte datasets, LEU33 and LEU37, were used to measure the transcript abundances of different leukocyte cells from healthy human PWB. For each dataset, the gene expression profiles of human healthy leukocyte subtypes were divided into two groups: one group profiled myeloid cells, including monocytes, neutrophils and eosinophils, while the other group profiled lymphoid cells, including T cells, NK cells and B cells. The average purity of each isolated cell subset was at least 92% as assessed by flow cytometry [20]. In Table 1, “Case” refers to the myeloid group, while “Control” refers to the lymphoid group.

### Detection of DE genes

The two-sample *t*-test method was used to identify DE genes by controlling the false discovery rate (FDR) [21] at 5%. Within the datasets, a DE gene was considered up-regulated if its relative difference of expression levels between the Case and Control group was larger than zero, and a DE gene was considered down-regulated if its relative difference of expression levels between the Case and Control group was smaller than zero [22]. Three types of DE genes were defined: DE genes between lung cancer and healthy controls, DE genes between inflammation-associated pulmonary diseases and controls and DE genes between myeloid cells and lymphoid cells.

### Evaluation of the consistency between two lists of DE genes

For two datasets, if a DE gene detected from one dataset was also identified as a DE gene with the same direction of regulation (up- or down-regulation) in another dataset, this gene was considered consistent across the datasets. We defined a consistency score as the percentage of consistent DE genes in all of the overlapping DE genes between two datasets. When comparing DE genes from different datasets generated on different platforms, we only considered the genes measured in both platforms. Then, using the binomial distribution model, we tested whether the consistency score of DE genes across datasets could be expected to occur by random chance. The probability of observing at least *m* DE genes each with same direction of regulation across two datasets from *N* randomly selected genes was calculated as follows: 
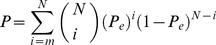
(1)in which *P_e_* is the random probability (here 0.5) of one DE gene having the same direction of regulation across two datasets. A consistency score was considered significant for p-value <0.05.

### Estimation of the proportions of myeloid cells and lymphoid cells in PWB

To determine whether the myeloid and lymphoid cell proportions differ in the PWB of pulmonary disease patients, we quantified the proportions of myeloid cells and lymphoid cells documented in the Immune Response in Silico (IRIS) database [23] by a process of deconvolution [24]. If *B* represents the known matrix of microarray expression profiles measured for a disease, comprising both disease and healthy samples; *X* represents the proportions of myeloid cells and lymphoid cells; and *A* represents the known matrix of expression levels of marker genes in the myeloid cells and lymphoid cells characterized by the IRIS database, then
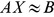
(2)


The object of deconvolution is to find the solution of the convolution equation, which will give the cell-type proportions for myeloid cells and lymphoid cells.

After the proportions of myeloid cells and lymphoid cells in each sample of a dataset were calculated by the Bioconductor package CellMix [25], we used the two-sample *t*-test method to evaluate whether the proportions were significantly different between disease and healthy controls. A p-value <0.05 was considered significant. Two other cell-specific expression signatures, defined by Abbas et al. [24] and by HaemAtlas [26], were also used to assess whether the proportion changes of myeloid cells and lymphoid cells were reproducible.

### DE genes for disease PWB defined by difference between myeloid and lymphoid cells

Blood cells can be grouped into myeloid cells and lymphoid cells. Therefore, for a gene in a PWB sample, the expression could be modelled as a linear combination of the expression of that gene in myeloid cells and lymphoid cells respectively. Let the average expression levels of a gene in myeloid cells and lymphoid cells be *b_m_* and *b_l_* respectively, then its expression level in healthy PWB sample can be modelled as
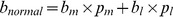
(3)where *p_m_* and *p_l_* represent the proportion of myeloid cells and lymphoid cells, respectively. When the proportion of myeloid cells increases with Δ*k* under disease condition, the proportion of lymphoid cells will decrease by Δ*k*. Thus, the expression level of the gene in the disease PWB sample can be represented as
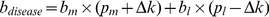
(4)


The expression difference of this gene between disease and healthy sample is
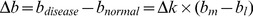
(5)


Based on the hypothesis that the shifted proportions of myeloid cells and lymphoid cells are the main factor contributing to the differential expression levels observed in disease PWB samples, according to formula (5), the direction of regulation (up- or down-regulation) of a DE gene in disease PWB samples compared to healthy PWB samples should be consistent with its direction of regulation in myeloid cells compared to lymphoid cells.

## Results

### Reproducibility of DE genes between lung cancer patients and healthy control subjects

To determine whether mRNA biomarkers for lung cancer can be reproducibly identified using PWB, we accessed data from three different microarray experiments. When comparing genes from multiple datasets generated on different platforms, we only considered the genes represented in all of the datasets (Table S1 in File S1). As shown in Table 2, with an FDR <5%, the DE genes detected from different datasets for lung cancer were highly reproducible. For example, among the 2029 DE genes identified from the LC46 dataset, 81.5% (1654) were also detected as DE genes in the LC60 dataset, and each of them had the same direction of regulation across the two datasets, which were derived from the same study. When comparing the DE genes identified from LC60 to the DE genes identified from LC153 which were from a different study, 389 DE genes were commonly detected, among which 99.2% had the same directions of regulation across the two datasets. Similarly, 258 DE genes overlapped between the 1372 and 876 DE genes respectively identified from LC46 and LC153, and 98.8% of them had the same directions of regulation. Based on the consistency scores, the overlap between each pair of lung cancer datasets could not be expected to have arisen by random chance (p-value <2.2×10^−16^, binomial test), indicating that the DE genes identified from PWB for lung cancer were significantly reproducible.

**Table 2:** Consistency of DE gene lists for lung cancer.

Dataset1	Dataset2	DE1^a^	DE2^b^	Overlapping DE^c^	Consistent DE^d^	Consistency score	Binomial P
LC60	LC46	4078	2029	1654	1654	100%	<2.2×10^−16^
LC60	LC153	2755	876	389	386	99.2%	<2.2×10^−16^
LC46	LC153	1372	876	258	255	98.8%	<2.2×10^−16^

a The number of DE genes identified from dataset1;

b The number of DE genes identified from dataset2;

c The number of overlapping DE genes;

d The number of consistent DE genes.

### Source of DE genes observed in lung cancer PWB samples

**Figure 1:**
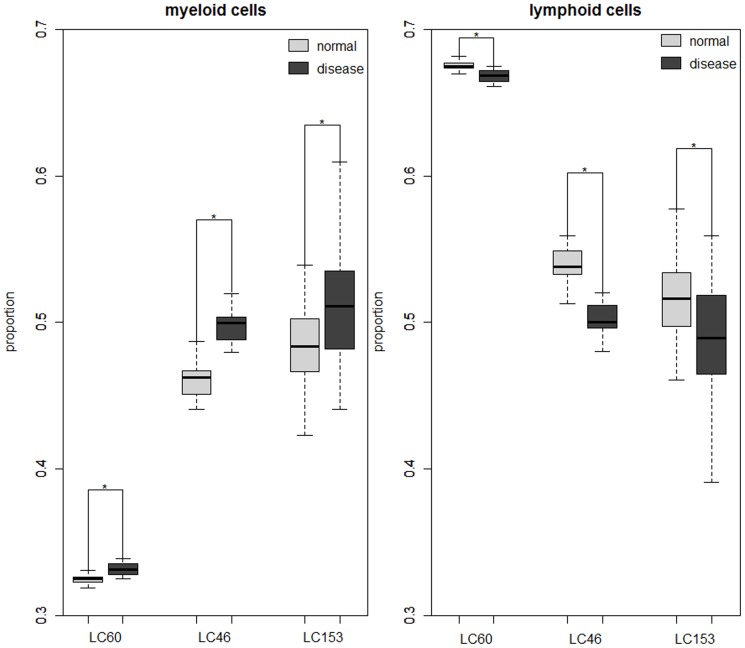
Boxplot of proportions of myeloid and lymphoid cells in lung cancer and control PWB samples. The estimated proportions of myeloid and lymphoid cells in lung cancer and healthy PWB samples for each dataset. * denotes statistically significant differences (P<0.05). Abbreviations are same as in Table 1.

To determine whether differences in the DE genes identified from different datasets might be explained by different proportions of myeloid cell and lymphoid cell subsets, we applied the gene expression deconvolution method [24] to estimate the proportions of the myeloid cells and lymphoid cells in each dataset of lung cancer and healthy control samples using the cell-specific signatures documented in the IRIS database (see Methods). As shown in Fig. 1, the proportions of myeloid cells were significantly higher in PWB samples with lung cancer compared to the healthy controls, while the proportions of lymphoid cells were significantly lower in lung cancer patients (p-value <0.05, *t*-test). Because the estimation depends on the selection of cell-type specific markers, we also used the cell-specific signatures defined by Abbas et al. [24] and the marker genes characterized by HaemAtlas [26] for the deconvolution. The results also showed that the estimated proportions of myeloid cells and lymphoid cells were significantly higher and lower respectively in PWB samples with lung cancer compared to the healthy controls (p-value <0.05, *t*-test).

To further analyze whether DE genes observed in lung cancer PWB samples might be defined by differential mRNAs between myeloid cells and lymphoid cells, we compared the DE genes identified between lung cancer and healthy controls with the DE genes identified in myeloid cells compared to lymphoid cells. We defined a reliable list of DE genes for lung cancer and for myeloid cells respectively. The list of DE genes for lung cancer was defined as the DE genes detected in all of the three datasets with consistent directions of regulation, which included 190 DE genes (Table S2 in File S1). To include as many DE genes between myeloid cells and lymphoid cells as possible, we defined the DE genes for myeloid cells compared to lymphoid cells by combining two lists of DE genes identified from two leukocyte datasets and deleting those DE genes with inconsistent directions of regulation across the two datasets. The resulting list included 5042 DE genes (referred to as the M-L DE gene list for simplicity). The integrated M-L DE gene list was reliable, as the DE genes identified from the two leukocyte datasets were significantly reproducible: using an FDR<5%, 5069 and 7266 DE genes between myeloid cells and lymphoid cells were identified, respectively, from the LEU33 and LEU37 datasets, and 4186 of the DE genes were included in both lists, 97.1% of which had the same directions of regulation across the two datasets, which was unlikely to be observed by random chance (p-value <2.2×10^−16^, binomial test).

Among the 190 DE genes consistently identified in lung cancer, 94.7% were included in the M-L DE genes list and all of them had the same directions of regulation as in myeloid cells versus lymphoid cells, which could not be expected to occur by random chance (p-value <2.2×10^−16^, binomial test). This indicates that the DE genes specific to lung cancer samples are predominately determined by population shifts in myeloid cells and lymphoid cells and mainly reflect the expression difference between these two types of cell. However, 10 of the 190 DE genes defined for lung cancer were not included in the M-L DE gene list, probably due to the incompleteness of the list of DE genes between myeloid cells and lymphoid cells, which was only derived from two datasets [27]. As evidence for this possibility, we further evaluated whether these 10 DE genes had the tendency of differential expression in any of the two leukocyte datasets. We found that, 4 of the 10 DE genes defined for lung cancer tended to be significantly expressed (with an unadjusted p-value <0.05) between myeloid cells and lymphoid cells and all of them had the same directions of regulation as in myeloid cells compared to lymphoid cells, which was unlikely to happen by random chance (p-value <2.2×10^−16^, binomial test). When relaxing the unadjusted p-value to 0.1, 6 of the 10 DE genes defined for lung cancer were DE genes between myeloid cells and lymphoid cells with the same directions of regulation. Notably, the DE genes from the lung cancer datasets with the most significant differences are more likely to be defined by differential mRNAs between myeloid cells and lymphoid cells. All of the top 10 most significantly DE genes defined for lung cancer were included in the M-L DE gene list, and all of them had the same directions of regulation as in the myeloid cell versus lymphoid cell datasets. Among the most significant top 100 DE genes defined for lung cancer, 96 had significantly different expression in myeloid cells and lymphoid cells, all with the same directions of regulation as in myeloid cells compared to lymphoid cells (p-value <2.2×10^−16^, binomial test). These results further suggested that the myeloid/lymphoid population shift was likely to constitute the source of the DE genes from lung cancer patients compared to healthy controls.

### Source of DE genes observed in PWB of inflammation-associated pulmonary diseases

Because the response of immune cells to inflammation could be represented by shifts in the blood cell populations [14], we also compared the DE genes identified from various inflammation-associated pulmonary diseases to the DE genes defined for myeloid cells. Based on the reproducibility analysis of three PWB gene expression datasets for each of the inflammation-associated pulmonary diseases (see Methods), the DE genes identified from different datasets for each inflammation-associated pulmonary disease were significantly reproducible (Table 3).

**Table 3:** Consistency of DE gene lists for each inflammation-associated pulmonary disease.

Dataset1	Dataset2	DE1^a^	DE2^b^	Overlapping DE^c^	Consistent DE^d^	Consistency score	Binomial P
SCD68	SCD55	2734	2336	1467	1467	100%	<2.2×10^−16^
SCD68	SCD58	2309	1749	740	738	99.7%	<2.2×10^−16^
SCD55	SCD58	1867	1749	587	581	99.0%	<2.2×10^−16^
TB63	TB54	3859	18919	3014	2982	98.9%	<2.2×10^−16^
TB63	TB83	3317	1602	773	771	99.7%	<2.2×10^−16^
TB54	TB83	11504	1602	1160	974	84.0%	<2.2×10^−16^
PNU58	PNU46	4179	4117	2828	2828	100%	<2.2×10^−16^
PNU58	PNU26	3474	208	74	54	73.0%	4.81×10^−5^
PNU46	PNU26	3292	208	82	60	73.2%	1.62×10^−5^

a The number of DE genes identified from dataset1;

b The number of DE genes identified from dataset2;

c The number of overlapping DE genes;

d The number of consistent DE genes.

For each of the three inflammation-associated pulmonary diseases, we also defined a reliable list of DE genes. With an FDR<5%, 441 genes that were significantly differentially expressed in all three sarcoidosis datasets with the same directions of regulation were defined as DE genes for sarcoidosis. Similarly, 550 and 34 genes were defined as DE genes for tuberculosis and pneumonia, respectively. Among the 441 DE genes defined for sarcoidosis, 90.2% (398) overlapped with the M-L DE genes and 97.0% of them had the same directions of regulation with respect as in the myeloid cells versus lymphoid cells. The number of DE genes overlapping between tuberculosis DE genes and M-L DE genes was 499, among which 97.0% were consistent in their directions of regulation. In pneumonia DE genes, 91.2% (31) of the 34 DE genes were included in the M-L DE gene list, and only one gene had an inconsistent directions of regulation in the M-L DE gene list. All of the consistency scores suggested that the data could not be observed by random chance (p-value <0.05, binomial test).

**Figure 2:**
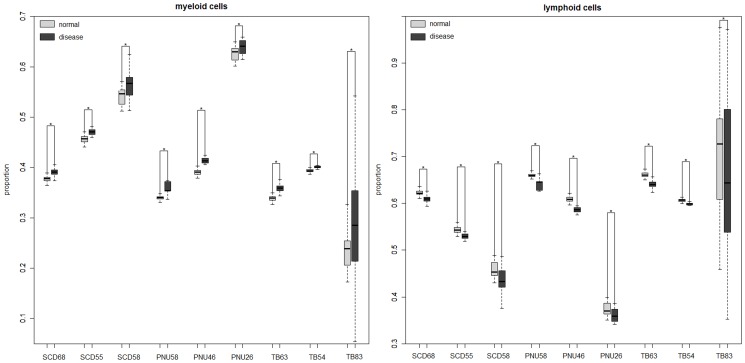
Boxplot of proportions of myeloid and lymphoid cells in inflammation-associated pulmonary disease and control PWB samples. The estimated proportions of myeloid and lymphoid cells in sarcoidosis, pneumonia and tuberculosis and healthy PWB samples for each dataset. * denotes statistically significant differences (P<0.05). Abbreviations are same as in Table 1.

Deconvolution of gene expression profiles also verified that the proportions of myeloid cells and lymphoid cells in the datasets for inflammation-associated pulmonary diseases changed, with the proportions of myeloid cells significantly increased and the proportions of lymphoid cells significantly decreased in inflammation-associated pulmonary disease samples compared to healthy controls (Fig. 2). This suggested that the observed differential gene expressions in the PWB transcriptome of inflammation-associated pulmonary diseases also tended to be overwhelmingly defined by differential mRNAs between myeloid cells and lymphoid cells.

### Comparison between lung cancer and inflammation-associated pulmonary diseases

As described above, the DE genes observed in both cancer and inflammation-associated pulmonary diseases tended to be predominantly originated from shifted populations of myeloid and lymphoid cells, suggesting that the DE genes might be consistent between them. Consequently, we compared the consistent DE genes defined for lung cancer to the consistent DE genes defined for each inflammation-associated pulmonary disease. For the 190 DE genes defined for lung cancer, 75, 104 and 7 were included in the 441, 550 and 34 DE genes defined for sarcoidosis, pneumonia and tuberculosis, respectively. All of them had the same directions of regulation with respect to their directions in the corresponding inflammation-associated pulmonary diseases, which could not be observed by random chance (p-value <0.05, binomial test). Then, we directly compared DE genes detected between lung cancer and each inflammation-associated pulmonary disease respectively. With an FDR<5%, few DE genes were identified between lung cancer and each inflammation-associated disease. Only one DE gene was commonly identified between lung cancer and sarcoidosis samples from GEO series GSE42826 and GSE42830, from which 28 and 30 DE genes were identified respectively. Five and 94 DE genes were identified between lung cancer and tuberculosis samples from GEO series GSE42826 and GSE42830 respectively, which shared only one gene. Extremely, no DE genes were identified between lung cancer and pneumonia samples from GEO series GSE42830. These results verified that the DE genes in lung cancer and inflammation-associated pulmonary diseases were likely to be defined by their differing cell populations rather than revealing differences in the disease conditions.

## Discussion

The selection of candidate blood-based mRNA biomarkers among the most significant DE genes in cancer blood samples compared to controls is a common strategy [28]–[30]. However, our results suggested that this strategy for distinguishing lung cancer from inflammation-associated pulmonary diseases may yield misleading results. Our results showed that DE genes detected from several PWB datasets for lung cancer and inflammation-associated pulmonary diseases were predominantly determined by subpopulation shifts in myeloid and lymphoid cells and mainly reflected the expression difference between myeloid cells and lymphoid cells. The comparison between the DE genes consistently identified from lung cancer and from each inflammation-associated pulmonary disease further showed that the overlapping DE genes in PWB samples of patients from these two groups of diseases were highly consistent in direction of regulation. Our results also showed that in PWB samples for lung cancer compared to healthy controls, the highest-ranking DE genes were most likely to be determined by the expression difference between myeloid cells and lymphoid cells, reflecting population shifts in myeloid cells and lymphoid cells. Because PBMCs include lymphocytes (T cells, B cells and NK cells) and monocytes, the DE genes observed in PBMCs of tumour patients could also mainly reflect shifted subpopulations of myeloid cells and lymphoid cells. Therefore, reported blood-based biomarkers by comparing gene expression profiles for cancer patients and healthy controls [4], [30]–[32] may have reduced power in distinguishing cancer from inflammation-associated disease because they are defined by DE genes between myeloid cells and lymphoid cells which will cause similar expression changes in inflammation-associated pulmonary diseases.

On the other hand, though the directions of regulation of DE genes in lung cancer and inflammation-associated pulmonary diseases versus healthy controls were almost the same, we could not exclude the possibility that the extent of subpopulation shifts in myeloid and lymphoid cells may be different between lung cancer and inflammation-associated pulmonary disease patients, which may cause subtle difference of gene expression between cancer and inflammation-associated pulmonary diseases. Bloom et al. have identified 144 genes that could distinguish tuberculosis from lung cancer [6]. Among these 144 genes, 59 were included in the genes analyzed in our study. We found that 47 of the 59 genes were detected as significant between myeloid cells and lymphoid cells with an FDR<5%, indicating that this 144-gene signature was likely to be influenced by the shifted populations of myeloid and lymphoid cells. As the signature was reported to be able to distinguish lung cancer from tuberculosis, this result may also hint that differences could exist in the extent of subpopulation shifts between lung cancer and inflammation-associated diseases. Considering the strong and similar influence of subpopulation shifts in PWB myeloid and lymphoid cells on the expression changes in cancer and inflammation-associated diseases, we suggested that an appropriate study design for finding cancer-specific diagnostic biomarkers might be to compare both the subpopulation shifts in myeloid cells and lymphoid cells and gene expression profiles between cancer and inflammation-associated diseases.

Another possibility is that subsets of peripheral blood cells may exhibit different gene expression patterns between healthy and disease states of cancer [33]. Actually, Showe and colleagues have reported that a 29-gene signature identified from PBMCs was promising in distinguishing lung cancer from nonmalignant pulmonary disease [3]. Though lack of validation in independent studies [2], this signature may suggest the feasibility of identifying cancer specific biomarkers from PWB cell subtypes as PBMCs are mainly composed of lymphocytes. Recent studies have also demonstrated that some interferon-stimulated genes (ISGs) are significantly down-regulated in blood T cells and B cells of patients with melanoma, breast cancer and gastrointestinal cancer [34], [35]. Conversely, ISGs tend to be significantly up-regulated in patients with inflammation-associated diseases such as SLE [36], which suggests a possible strategy for distinguishing disease types. We have explored the potential of this strategy using two datasets that include subsets of lymphocytes from SLE and healthy control blood (Table S3 in File S1). From the 190 DE genes defined for lung cancer, we obtained two genes that were least likely to be differentially expressed between myeloid and lymphoid cells (with an unadjusted p-value>0.2), one of which was significantly down-regulated in the B cells and CD4 T cells from SLE samples compared to healthy controls (Table S4 in File S1). This suggest that future identification of biomarkers from tumour PWB samples might be developed through comparing cancer and inflammation cell subsets directly.

## Supporting Information

File S1 **Table S1-S4.** Table S1. Number of genes shared between different platforms; Table S2. The 190 DE genes defined for lung cancer; Table S3. Datasets of systemic lupus erythematosus; Table S4. Cell type specific expression in systemic lupus erythematosus.
(XLS)
